# *ACTA1*‐Related Adult‐Onset Scapuloperoneal Myopathy With Cores and Rods

**DOI:** 10.1111/nan.70067

**Published:** 2026-03-13

**Authors:** Alexandru Caramizaru, Marion Onnée, Sergey Nikitin, Amelia Dobrescu, Gianmarco Severa, Aysylu Murtazina, Andoni Urtizberea, Jean‐Pascal Lefaucheur, Robert‐Yves Carlier, Corinne Metay, Edoardo Malfatti

**Affiliations:** ^1^ Regional Center for Medical Genetics Dolj Craiova Romania; ^2^ Medical Genetics Department University of Medicine and Pharmacy of Craiova Craiova Romania; ^3^ University Paris Est Créteil, Inserm, U955, IMRB Créteil France; ^4^ Research Center for Medical Genetics Moscow Russia; ^5^ Reference Center for Neuromuscular Disorders, APHP Henri Mondor University Hospital France; ^6^ Institut de Myologie Paris France; ^7^ Excitabilité Nerveuse et Thérapeutique, Faculté de Médecine de Créteil Université Paris‐Est‐Créteil, Hôpital Henri Mondor Créteil France; ^8^ Service de Physiologie‐Explorations Fonctionnelles, Hôpital Henri Mondor Créteil France; ^9^ AP‐HP, GHU Paris‐Saclay, DMU Smart Imaging, Medical Imaging Department Raymond‐Poincaré Teaching Hospital Garches France; ^10^ UMR 1179 End:Icap, UVSQ‐Paris‐Saclay University Paris France; ^11^ APHP, Centre de Génétique Moléculaire et Chromosomique, Service de Biochimie Métabolique, U. F de Cardiogénétique et Myogénétique Moléculaire et Cellulaire, Groupe Hospitalier La Pitié‐Salpêtrière Charles Foix Paris France; ^12^ INSERM UMRS 974, Sorbonne University, Institute of Myology Paris France

**Keywords:** *ACTA1*, actinopathy, cardiac alpha‐actin, cores, rods, scapuloperoneal myopathy

## Abstract

**Aims:**

Actinopathies are myopathies associated with pathogenic variants in *ACTA1*, a gene encoding the skeletal alpha‐actin protein. Although patients most frequently have a severe congenital myopathy, an important clinical and myopathological variability has been described. Recently, a scapuloperoneal myopathy phenotype associated with *ACTA1* has been reported. Here, we present a Russian woman with a late‐onset, slowly progressive, scapuloperoneal actinopathy associated with an unpublished heterozygous pathogenic *ACTA1* variant.

**Methods:**

We performed a thorough analysis of clinical, muscle imaging, muscle biopsy, genetic, protein and cardiac alpha‐actin expression data from a 65‐year‐old woman with a scapuloperoneal myopathy phenotype.

**Results:**

Disease onset was at around 30 years with proximal lower limbs muscle weakness, which slowly progressed towards an upper and lower limb distal involvement with prominent weakness of the fourth and fifth finger extensors. A muscle MRI showed a symmetric axial involvement, while lower limbs sections evidenced a severe symmetric involvement of *quadriceps* and *biceps femoris* long head, and a symmetric involvement of medial *gastrocnemius* associated with a right *tibialis anterior* involvement. Muscle biopsy showed cores and rods. The patient harboured the unpublished NM_001100.4:c.1001C > T, p.(Pro334Leu) *ACTA1* variant. Immunofluorescence and western blot studies showed an increased expression of cardiac alpha‐actin, an actin isoform which is normally predominant in the prenatal skeletal muscles and adult heart muscle, suggesting a possible role of this isoform in milder actinopathy phenotypes.

**Conclusions:**

We report a milder, late‐onset, slowly progressive scapuloperoneal myopathy phenotype with cores and rods and cardiac alpha‐actin overexpression, thus expanding the spectrum of actinopathies.

## Summary

- Actinopathies are clinically, genetically, and myopathologically heterogeneous myopathies associated with pathogenic variants in *ACTA1*, a gene encoding skeletal alpha‐actin.
- A scapuloperoneal phenotype related to *ACTA1* has been previously described in one family.
- We report a patient with an adult‐onset, slowly progressive, scapuloperoneal myopathy with cores and rods, harbouring the *ACTA1* NM_001100.4:c.1001C > T, p.(Pro334Leu) heterozygous variant.
- The scapuloperoneal phenotype could represent a distinct clinical subcategory, and the characterisation of this patient with a less severe, different clinical presentation and cores and rods contributes to its understanding.

## Graphical abstract

We report a patient with an adult‐onset, slowly progressive, *ACTA1*‐related scapuloperoneal myopathy with cores and rods, determined by the heterozygous variant NM_001100.4:c.1001C > T, p.(Pro334Leu). The scapuloperoneal phenotype could represent a distinct subcategory, and the characterisation of this patient with a less severe, different clinical presentation and nemaline bodies should contribute to its understanding.

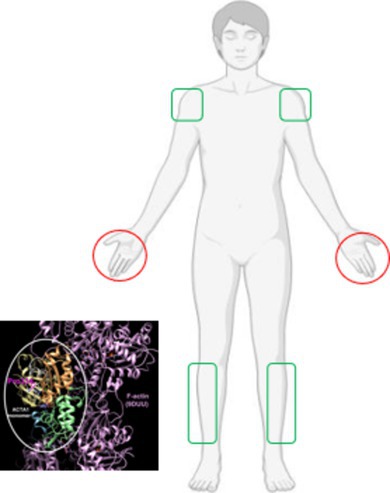

The *ACTA1* gene (1q42.13) encodes skeletal muscle alpha‐actin, a key contractile protein belonging to the actin family. Skeletal and cardiac alpha‐actin are the two sarcomeric isoforms expressed in muscle [1]. During foetal development, cardiac alpha‐actin predominates, but skeletal alpha‐actin becomes the main isoform by birth, while the postnatal heart primarily expresses cardiac alpha‐actin [2]. Cardiac involvement in actinopathies is rare [3, 4, 5], and even severe lethal cases usually allow survival to term [2]. Increased cardiac alpha‐actin expression in some long‐term survivors [6] suggests a compensatory role, mitigating the effects of *ACTA1* variants [7].

Actinopathies are typically autosomal dominant [8] and clinically heterogeneous, ranging from severe congenital to milder adult‐onset forms [9]. Muscle biopsies show overlapping lesions, including actin accumulations, rods (intranuclear in 6% of cases), fibre‐type disproportion, cores, caps, dystrophic features, zebra bodies and lobulated fibres [3, 10]. Nemaline bodies (rods), derived from the Z‐line, are the hallmark of nemaline myopathy (NM) [11]. NM is most often caused by variants in *NEB*, encoding nebulin, a thin‐filament associated protein [12, 13, 14], and *ACTA1*, accounting for 50% and 20% of all NMs, respectively [15, 16].

*ACTA1* has recently been linked to a novel scapuloperoneal myopathy phenotype, observed in 14 individuals within a single family [17]. This presentation features early scapuloperoneal and distal weakness, followed by mild facial involvement, Achilles tendon contractures and diminished reflexes, broadening the clinical spectrum of actinopathies.

Here, we report a patient with an adult‐onset slowly progressive scapuloperoneal myopathy related to a novel heterozygous pathogenic *ACTA1* variant, NM_001100.4:c.1001C > T, p.(Pro334Leu), thus enlarging the landscape of *ACTA1*‐related disorders.

A 65‐year‐old woman, born to nonconsanguineous Russian parents, with no relevant family or early medical history, developed motor symptoms at 30, beginning with running difficulties and a stepping gait. By 48, she experienced progressive weakness affecting stair climbing, rising from a chair and bilateral finger extension (fourth–fifth digits). At 50, frequent falls occurred due to thigh weakness, especially when descending stairs.

Neurologic examination at 50 revealed right fourth finger extensor and quadriceps weakness (MRC 4), axial and mild distal leg weakness, thigh and gluteal atrophy, and reduced or absent reflexes, without sensory deficits. Serum CK was 360 U/L.

At 63, she showed a right‐predominant stepping gait, bilateral asymmetric scapula alata and limited lateral gaze, without other cranial involvement. Muscle testing demonstrated neck flexor (MRC 4) and proximal deltoid (MRC 4) weakness, severe extensor weakness for the fourth and fifth fingers bilaterally (MRC 2, Figure S1), and symmetric proximal–distal lower limb weakness involving the *psoas*, *quadriceps* and *tibialis anterior* (MRC 4).

A whole‐body muscle MRI, performed at the age of 64 (Figure S2), highlighted a predominant involvement of the tongue, *quadriceps*, *soleus* and *tibialis anterior*, with relative sparing of the *gracilis* and select hamstring muscles.

A deltoid muscle biopsy at 63 years of age, showed the presence of clusters of cytoplasmic rods indicated by yellow arrowheads, and cores in separate areas of the myofibres, indicated by white asterisks (Figure 1A). Oxidative histoenzymatic reaction NADH‐TR revealed the presence of both intermyofibrillar disorganisation (yellow asterisk) and well‐delimited cores (black asterisk, Figure 1B). There was a type 2 fibre predominance. Transmission electron microscopy ultrastructural studies confirmed the presence of Z‐line thickening forming typical rods (Figure 1C), characterised by orthogonal net disposition of filaments and the presence of well‐defined areas of disorganisation and Z‐line material accumulation, corresponding to typical, unstructured cores; there are no mitochondria visible in the core area (Figure 1D).

**FIGURE 1:**
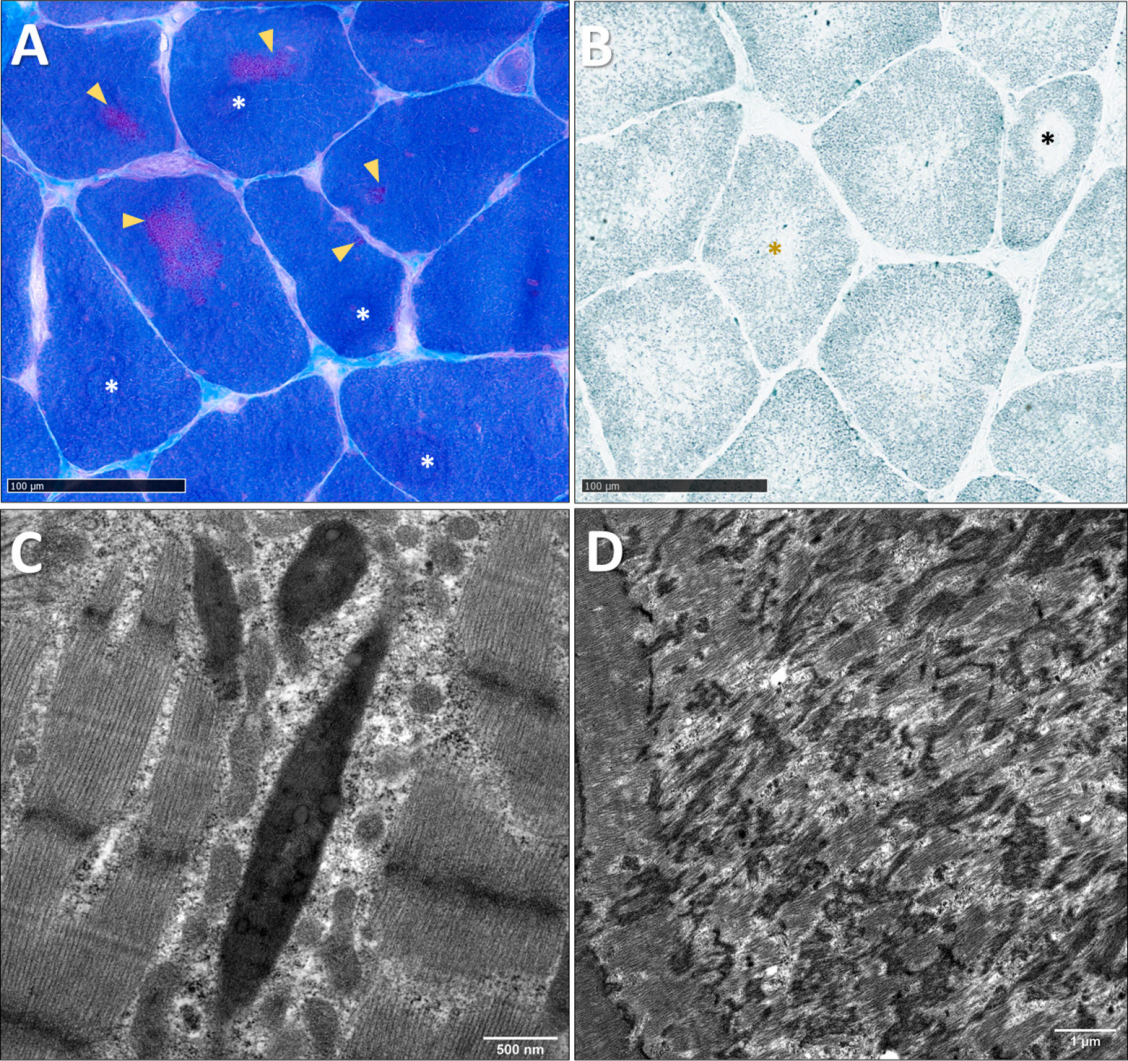
Muscle morphological studies. (A,B) Light microscopy. (C,D) Electron microscopy. (A) Masson trichrome staining showing the presence of clusters of rods appearing as fuchsinophilic granular material found in the cytoplasm (yellow arrowheads), in proximity but separated from cores (white asterisks). (B) NADH‐TR oxidative histoenzymatic reaction showing the presence of areas devoid of oxidative staining corresponding to cores (black asterisk), and intermyofibrillar disorganisation (yellow asterisk). (C) Electron microscopy showing the presence of Z‐line thickening forming typical rods characterised by orthogonal net disposition of filaments. (D) Electron micrograph showing well‐defined areas of disorganisation, Z‐line material accumulation, devoid of mitochondria, corresponding to a typical unstructured core.

A heterozygous missense variant was identified in exon 7 of *ACTA1* (NM_001100.4:c.1001C > T, p.(Pro334Leu)), characterised as Class 4, Likely Pathogenic according to the 2015 ACMG guidelines [18].

We performed cardiac muscle alpha‐actin (ACTC1) protein expression analysis (Figure 2) by immunostaining using anti‐ACTC1 antibody, highlighting an overexpression of cardiac alpha‐actin in the patient's biopsy compared to controls. This observation was confirmed by western blot analysis. We could also observe a significant fibre hypertrophy in the patient's muscle biopsy compared to the control.

**FIGURE 2:**
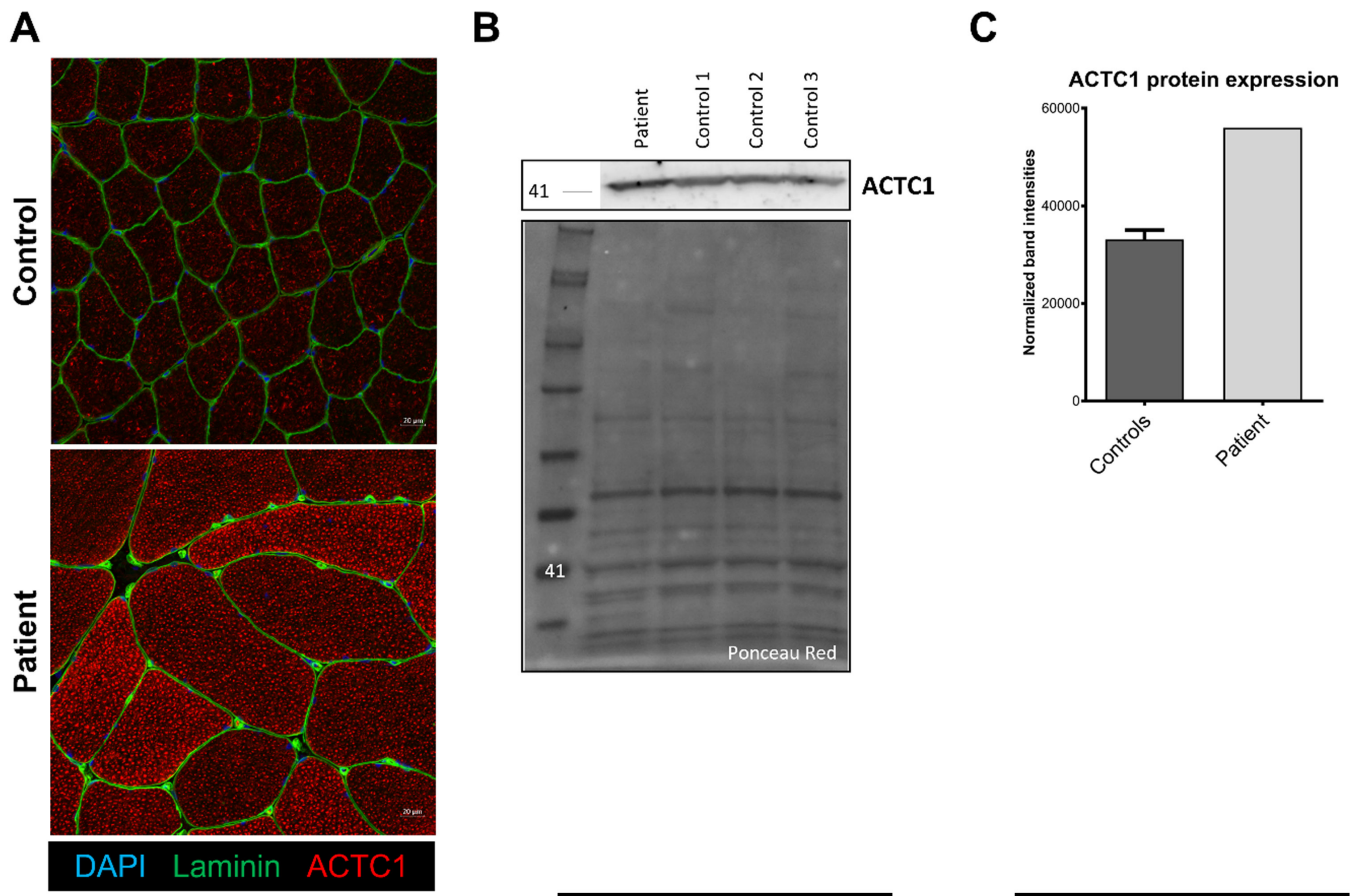
Analysis of cardiac muscle alpha‐actin (ACTC1) protein expression in deltoid muscle biopsies from patient and age‐ and sex‐matched controls. (A) Immunofluorescence staining of ACTC1 in red, nuclei are stained in blue and laminin in green. Scale bar: 20 μm. (B,C). Western blot analysis from muscle homogenates from patient and controls (B), and quantification (C). Ponceau red is used as the loading control.

Detailed results (clinical, MRI, biopsy, genetic, protein and cardiac alpha‐actin expression) are available in Table S1.

Actinopathies usually manifest as severe congenital myopathies with hypotonia, weakness, facial involvement and respiratory or feeding difficulties, though adult‐onset forms also occur [3]. Nemaline myopathies show similar variability [11], with *ACTA1* variants accounting for over half of severe cases [6]. Our patient's onset at 30 years is therefore atypical.

In the previously reported family with *ACTA1*‐related scapuloperoneal myopathy (c.591G > T, p.(Glu197Asp)) [17], disease onset and severity varied, with distal‐to‐proximal leg weakness and early scapular winging, deltoid/thenar atrophy, and distal finger extensor deficits,partially resembling our patient's finger extensor weakness. However, our patient showed initial proximal lower limb involvement followed by distal progression.

Other *ACTA1*‐related distal myopathies have similarly featured finger flexor and extensor weakness, particularly in digits 3–5, though onset and course differ among families. Therefore, such findings should be interpreted within the broader clinical and familial context when guiding diagnosis [19, 20].

MRI studies of *ACTA1*‐related myopathies show characteristic thigh involvement of the *vasti*, *sartorius* and *biceps femoris*, with relative sparing of the adductors and *gracilis*. In the lower legs, the *soleus* and *tibialis anterior* are typically affected, while the *gastrocnemii* and *tibialis posterior* remain relatively preserved [21, 22]. In the family with *ACTA1*‐related scapuloperoneal myopathy, one patient showed marked atrophy and significant fatty replacement of the *quadriceps*, while severe involvement was noted in the hamstring muscles, with partial sparing of *semitendinosus* and *adductor longus* muscles; a milder case showed minimal changes in the *sartorius*, *biceps femoris* and *vastus lateralis* [17]. Notably, fibrofatty tongue replacement may also occur in actinopathies without dysphagia [23], a rare finding outside disorders such as Duchenne muscular dystrophy, facioscapulohumeral dystrophy [24], oculopharyngeal muscular dystrophy [25] or late‐onset Pompe disease [26]. Our patient's MRI broadly reflected these patterns, highlighting notable interindividual variability.

*ACTA1* variants cause a spectrum of myopathies (including intranuclear rod, actin‐accumulation, cap myopathy and congenital fibre‐type disproportion) [27, 28, 29, 30], characterised by overlapping histological features such as actin aggregates, rods, caps and core‐like areas [3]. Severe neonatal cases often show type I fibre atrophy with rods, cores or cytoplasmic bodies [6]. Our patient's biopsy revealed rods and cytoplasmic cores, consistent with this morphopathologic variability, and also partly resembling core‐rod myopathy [31]; however, the clinical phenotype is not supportive of this diagnosis, although *ACTA1* variants have been linked to core and rod pathology [32, 33, 34].

Muscle pathology in *ACTA1*‐related scapuloperoneal myopathy ranges from marked fibre size variability, nuclear internalisation and fatty infiltration to mild changes without rods or cores. Notably, nemaline rods, cores or actin aggregates were absent in these cases [17], contrasting with our patient's biopsy.

A significant variability has also been reported in distal myopathies with finger weakness, which may show, among other changes, rods, cores or fibre‐type disproportion [19, 20, 35]. These findings are overall similar to the ones described in our patient.

Around 90% of *ACTA1* variants are autosomal dominant missense changes, while recessive cases typically result from null mutations [3]. The NM_001100.4:c.1001C > T (p.(Pro334Leu)) variant affects a conserved residue, is absent from population databases, and is predicted to be deleterious (REVEL 0.814; CADD 32) [36]. *ACTA1* shows low tolerance for benign missense variation, with pathogenic missense changes representing a common disease mechanism (ClinGen Congenital Myopathies VCEP ‐ Variant Curation Expert Panel). This variant has been previously reported in ClinVar, albeit with limited clinical context. Notably, a similar substitution at this codon (p.(Pro334Arg)) has been reported as likely pathogenic in a late‐onset dominant alpha‐actinopathy with consistent muscle biopsy findings (Invitae, Revvity Omics, CeGaT and Paris‐East Créteil University internal data, Alan Beggs personal communication, Leiden Open Variation Database). Accordingly, the evidence supports the classification of p.(Pro334Leu) as likely pathogenic under current ACMG/AMP criteria.

Variants near Pro334 (e.g., p.(Pro332Ser)) cause severe congenital fibre‐type disproportion with marked weakness and respiratory involvement but preserved ocular motility [37]; our patient's mild left‐gaze limitation therefore diverges from this pattern.

Since severe early‐onset phenotypes often correlate with variants in exons 2–5 [6], the milder, late‐onset presentation in our patient may reflect the variant's location in exon 7 [1]; however, reports of mild phenotypes with upstream variants [19, 20, 35] suggest that additional modifying factors likely contribute.

In survivors of severe *ACTA1*‐related nemaline myopathy beyond the neonatal period [6], increased cardiac alpha‐actin expression has been reported as a potential compensatory mechanism. Our patient similarly demonstrates cardiac alpha‐actin expression, possibly contributing to the milder phenotype. Although such upregulation is rarely described in thin‐filament myopathies outside *ACTA1* variants [38, 39], persistence of developmental proteins (including cardiac alpha‐actin) has been observed in severe congenital myopathies caused by *MYL1* and other thick‐filament gene defects, particularly with respect to myosin isoform expression [40]. These observations suggest that cardiac alpha‐actin expression may modulate disease severity in actinopathies.

With this patient, we expand the clinical and genetic spectrum of *ACTA1* myopathies. *ACTA1*‐related congenital myopathies remain a clinically, genetically and histopathologically variable group of disorders. Particularly, the scapuloperoneal phenotype could represent a distinct subcategory, and the characterisation of this patient with a less severe, different clinical presentation and nemaline bodies should contribute to its understanding.

## Author Contributions

E.M. designed the study. S.N., E.M., G.S. and M.O. performed the study and analysed the data. M.O. performed the experiments. S.N., E.M. and G.S. provided clinical data and provided resources. A.C., M.O., S.N. and G.S. wrote the manuscript. E.M. supervised the study. The manuscript was revised by E.M., C.M., R.C., J.L., A.U., A.D., A.M. and S.N.

## Funding

The authors have nothing to report.

## Ethics Statement

This study was approved by the ethical Committee issued by our institutions in compliance with the Declaration of Helsinki. Informed consent has been obtained from the patient for participation and muscle biopsy studies, according to the French Comité de Protection des Personnes Est IV DC‐2012‐1693.

## Consent

Informed consent for publication, including pictures, has been obtained from the patient.

## Conflicts of Interest

The authors declare no conflicts of interest.

## Supporting information


**Figure S1:** Weakness of the extensors of the fourth and fifth fingers of the hands.

**Figure S2:** Muscle imaging of multiple sequential T1W and STIR MRI acquisitions in the axial plan. CRANIAL sections (A, B): fibrofatty infiltration of tongue (A, arrow). AXIAL sections (C–F): focal left deltoid involvement at the medial side (C, arrow) and bilateral substitution of paraspinal muscle at the lumbar level (E, arrow). LOWER LIMBS sections (G–N): severe and bilateral fibrofatty substitution of *quadriceps* and *biceps femoris* long head (I, arrows) associated with *quadriceps* muscle hypersignal in STIR sequences (J, yellow asterisk). *Gastrocnemius medialis* and *tibialis anterior* involvement (K and M, arrows); *Soleus* hypersignal in STIR (N, yellow asterisk).

**Figure S3:** Two‐dimensional and three‐dimensional ACTA1 protein structure. (A) Localisation of *ACTA1* c.1001C > T, p.(Pro334Leu) variant is indicated in red on the *ACTA1* gene and protein subdomains. ‘SD’ indicates the different ACTA1 subdomains, which are colour‐coded to correspond to the colours seen throughout the 3D protein structure in Panel C. The hinge domains are represented in grey and correspond to amino acids 137 to 150 and 333 to 338. (B) The Pro334Leu missense variant is located in a highly conserved region among 11 different species. (C) ACTA1 is divided into two domains connected by two hinge domains; each ACTA1 domain is further divided into two subdomains (SD 1 and 2; and SD 3 and 4). Subdomains‐coloured model of ACTA1 monomer was built using AlphaFold Protein Structure Database (https://alphafold.ebi.ac.uk/; entry P68133). The residue 334 is shown in magenta (Pro334 in the left panel and mutated Leu334 in the right panel). The residues at < 5 Å of residue 334 are shown in stick representation with coloured atoms (H in white, O in red, and N in blue). Hydrophobic interactions are shown by yellow lines.

**Figure S4:** Cryo‐EM structure of recombinant wild‐type ACTA1 phalloidin‐stabilised F‐actin (light pink) (PDBID 9DUU on https://www.rcsb.org/). One ACTA1 monomer is coloured in yellow, blue, orange and green, representing its different subdomains (SD1, SD2, SD3 and SD4, respectively), the hinge domain is represented in light grey. The residue Pro334 is represented in magenta. We can notice that the Pro334 residue is in the outside part of the F‐actin, likely interacting more with other proteins of the thin filament rather than with other ACTA1 monomers.

**Table S1:** Detailed clinical, muscle MRI, muscle biopsy, genetic, protein and cardiac alpha‐actin expression data regarding a patient with an *ACTA1*‐related adult‐onset scapuloperoneal myopathy with cores and rods.

## Data Availability

All data generated or analysed during this study are included in this published article.
