# Supplementary figures and images for: ACTA1‐Related Adult‐Onset Scapuloperoneal Myopathy With Cores and Rods
Source: Neuropathol Appl Neurobiol. 2026 Mar 13;52(2):e70067. doi: 10.1111/nan.70067 (PMC12987713; doi:10.1111/nan.70067)

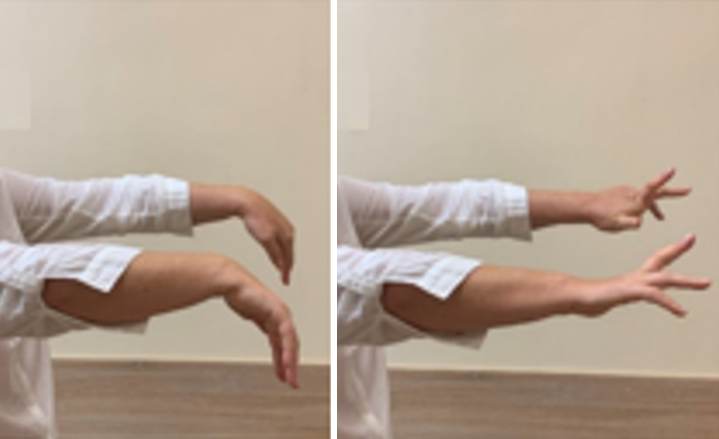

Supplement: Supplementary file 1 — Figure S1: Weakness of the extensors of the fourth and fifth fingers of the hands. [file NAN-52-e70067-s004.tiff]

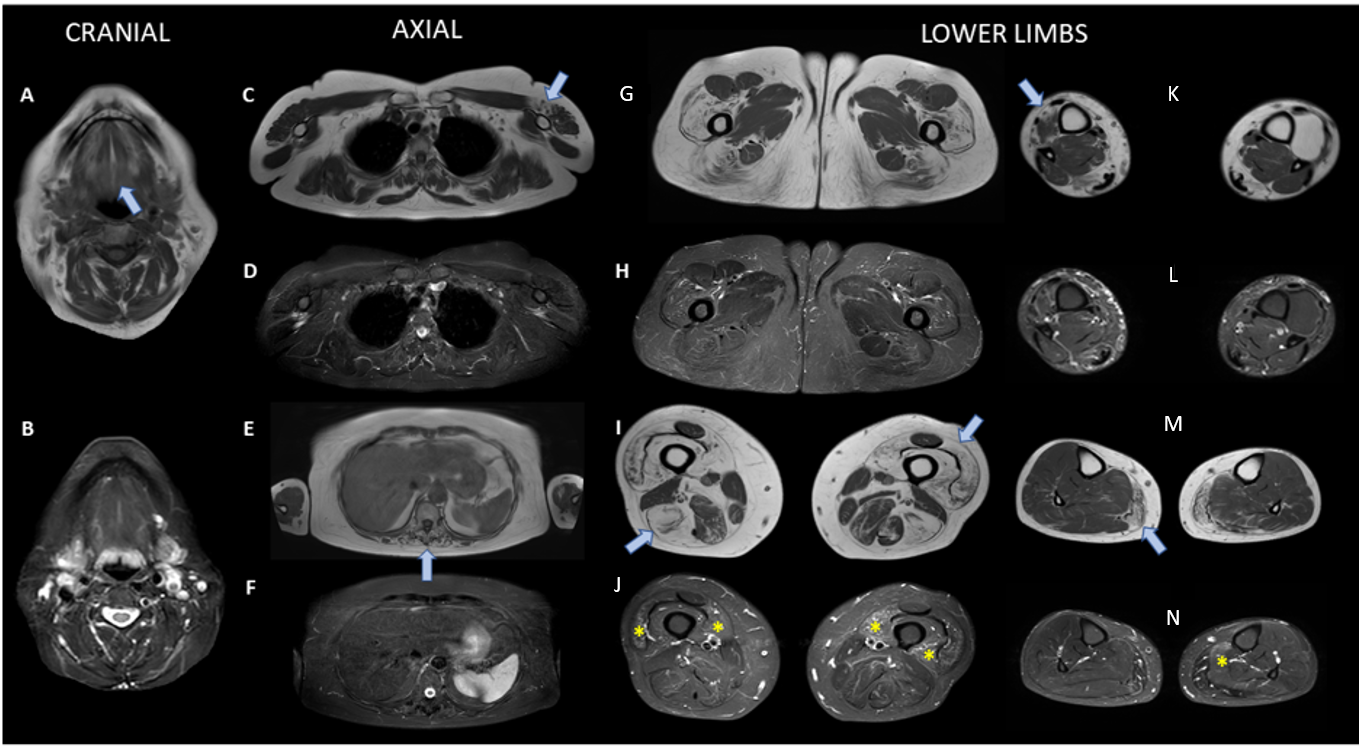

Supplement: Supplementary file 2 — Figure S2: Muscle imaging of multiple sequential T1W and STIR MRI acquisitions in the axial plan. CRANIAL sections (A, B): fibrofatty infiltration of tongue (A, arrow). AXIAL sections (C–F): focal left deltoid involvement at the medial side (C, arrow) and bilateral substitution of paraspinal muscle at the lumbar level (E, arrow). LOWER LIMBS sections (G–N): severe and bilateral fibrofatty substitution of quadriceps and biceps femoris long head (I, arrows) associated with quadriceps muscle hypersignal in STIR sequences (J, yellow asterisk). Gastrocnemius medialis and tibialis anterior involvement (K and M, arrows); Soleus hypersignal in STIR (N, yellow asterisk). [file NAN-52-e70067-s001.tiff]

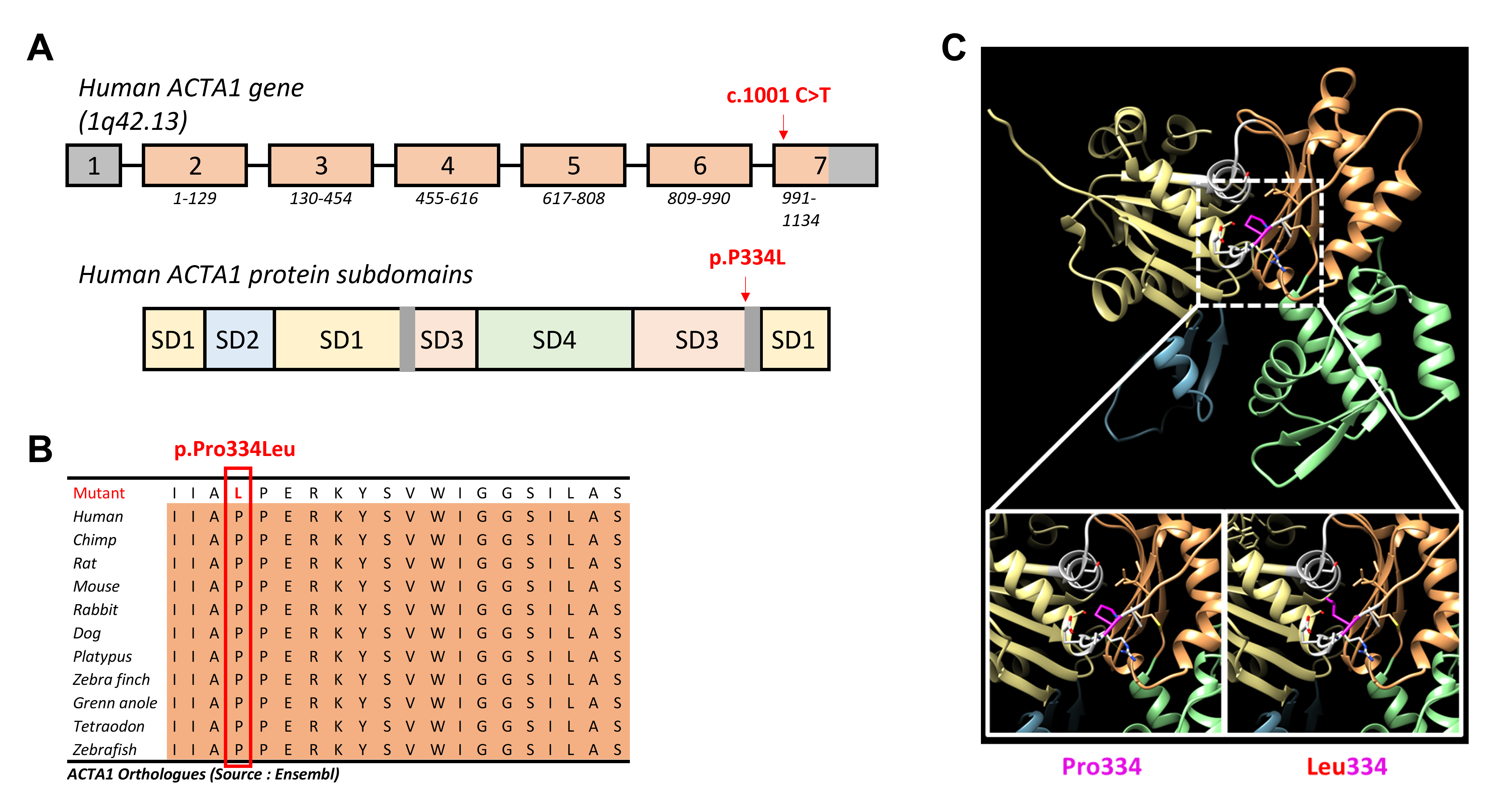

Supplement: Supplementary file 3 — Figure S3: Two‐dimensional and three‐dimensional ACTA1 protein structure. (A) Localisation of ACTA1 c.1001C > T, p.(Pro334Leu) variant is indicated in red on the ACTA1 gene and protein subdomains. ‘SD’ indicates the different ACTA1 subdomains, which are colour‐coded to correspond to the colours seen throughout the 3D protein structure in Panel C. The hinge domains are represented in grey and correspond to amino acids 137 to 150 and 333 to 338. (B) The Pro334Leu missense variant is located in a highly conserved region among 11 different species. (C) ACTA1 is divided into two domains connected by two hinge domains; each ACTA1 domain is further divided into two subdomains (SD 1 and 2; and SD 3 and 4). Subdomains‐coloured model of ACTA1 monomer was built using AlphaFold Protein Structure Database (https://alphafold.ebi.ac.uk/; entry P68133). The residue 334 is shown in magenta (Pro334 in the left panel and mutated Leu334 in the right panel). The residues at < 5 Å of residue 334 are shown in stick representation with coloured atoms (H in white, O in red, and N in blue). Hydrophobic interactions are shown by yellow lines. [file NAN-52-e70067-s002.tiff]

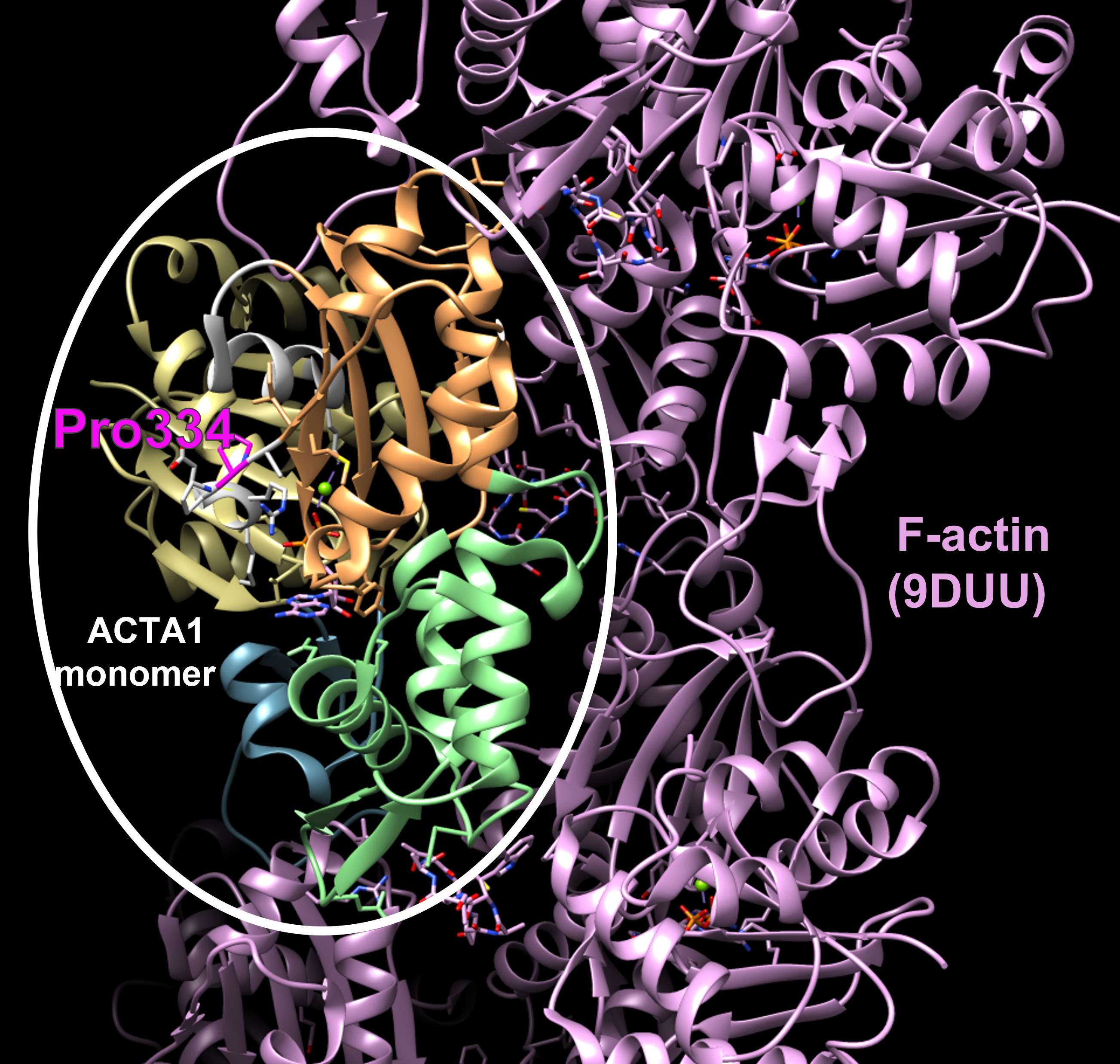

Supplement: Supplementary file 4 — Figure S4: Cryo‐EM structure of recombinant wild‐type ACTA1 phalloidin‐stabilised F‐actin (light pink) (PDBID 9DUU on https://www.rcsb.org/). One ACTA1 monomer is coloured in yellow, blue, orange and green, representing its different subdomains (SD1, SD2, SD3 and SD4, respectively), the hinge domain is represented in light grey. The residue Pro334 is represented in magenta. We can notice that the Pro334 residue is in the outside part of the F‐actin, likely interacting more with other proteins of the thin filament rather than with other ACTA1 monomers. [file NAN-52-e70067-s003.tiff]
