# Supplementary material for: ACTA1‐Related Adult‐Onset Scapuloperoneal Myopathy With Cores and Rods
Source: Neuropathol Appl Neurobiol. 2026 Mar 13;52(2):e70067. doi: 10.1111/nan.70067 (PMC12987713; doi:10.1111/nan.70067)
Supplement: Supplementary file 5 — Table S1: Detailed clinical, muscle MRI, muscle biopsy, genetic, protein and cardiac alpha‐actin expression data regarding a patient with an ACTA1‐related adult‐onset scapuloperoneal myopathy with cores and rods. [file NAN-52-e70067-s005.docx]

Supplementary Table 1. Detailed clinical, muscle MRI, muscle biopsy, genetic, protein and cardiac alpha-actin expression data regarding a patient with an *ACTA1*-related adult-onset scapuloperoneal myopathy with cores and rods.

| Evaluation | results |
| --- | --- |
| HISTORY | 65-year-old woman born to non-consanguineous Russian parents, with no reported family history of neuromuscular disease; she had a normal motor development and an active childhood/early adulthood. During schooling, she was no different from her peers in athletic endeavours, and at 25 years, she gave birth to a healthy daughter. |
| ClinicaL | At 30, she began to experience difficulties with running, followed by the development of a stepping gait. By 48, she noticed progressive challenges in climbing stairs and rising from a chair, associated with the onset of a slowly progressive bilateral weakness of the 4^th^ and 5^th^ fingers. At 50 years old, she developed frequent falls attributed to sudden thigh loss of strength, particularly when descending stairs.  The first neurologic examination, performed at 50 years, showed weakness of extensors of the 4^th^ finger of the right hand, quadriceps femoris weakness (4 MRC), and axial muscle weakness. There was mild weakness in the foot flexors/extensors as the patient could stand on toes but had difficulties standing on heels. The thigh and gluteal muscles showed atrophy. Deep tendon reflexes were diminished in the upper extremities and absent in the lower extremities. There were no sensory disturbances. The serum Creatine Kinase level was 360 U/L.  At the age of 63, a new evaluation revealed a stepping gait predominantly affecting the right foot. Additionally, the patient exhibited bilateral, asymmetrical *scapula alata*. There was no oculo-bulbo-facial involvement except for a non-specific limitation of the lateral gaze. Manual muscle testing revealed neck flexor weakness (4 MRC), proximal symmetric deltoid weakness (4 MRC), and a prominent upper limb distal muscle weakness, with the finger extensors at 2 MRC for the 4^th^ and 5^th^ fingers bilaterally (Supplementary Figure 1). In the lower limbs, there was a symmetric proximal-distal weakness of the psoas, quadriceps, and *tibialis* *anterior* muscles (4 MRC). |
| EMG | Diffuse myogenic alterations with low amplitude spontaneous activity in the *vastus lateralis*, lateral *gastrocnemius* and *extensor digitorum* muscles, without abnormal conduction velocities. |
| Muscle MRI | At the cranial level, a symmetric fibro-fatty infiltration of the tongue was noticed, with no alteration in the masticatory or cervical paraspinal muscles (Supplementary Figure 2A and B). A symmetric axial involvement was noticed with a moderate fibro-fatty infiltration of the paraspinal muscle at the lumbar level (Supplementary Figure 2E); focal involvement of the medial side of the left deltoid muscle was also found (Supplementary Figure 2C). Lower limbs sections revealed: a) at thigh level, a severe symmetric involvement of *quadriceps* and *biceps femoris* long head (Supplementary Figure 2G and 2I), with sparing of *rectus femoris, adductors, quadratus femoris, biceps femoris* short head, *gracilis*, and *sartorius*, and relative sparing of *semimembranosus* and *semitendinosus*; b) at leg level, a symmetric involvement of medial *gastrocnemius* associated with a right *tibialis anterior* involvement (Supplementary Figure 2K and 2M). In STIR sequences, a *quadriceps* and *soleus* hypersignal linked to muscle degeneration was noticed (Supplementary Figure 2J and 2N). |
| Muscle Biopsy | A deltoid muscle biopsy (at 63 years of age) showed the presence of clusters of cytoplasmic rods (Figure 1A) (indicated by the yellow arrowhead and the asterisk). Oxidative histochemical reactions revealed both intermyofibrillar disorganisation (blue asterisk) and well-delimited cores (black asterisk, Figure 1A and B). The rods and the cores were found in separate areas of the myofibers. There was a type 2 fibre predominance. Transmission electron microscopy studies confirmed the presence of Z-line thickening forming typical rods (Figure 1C) and the presence of well-defined areas of disorganisation devoid of mitochondria with Z-line material accumulation, corresponding to cores (Figure 1D). |
| Genetic | The *ACTA1* variant, NM_001100.4:c.1001C>T, p.(Pro334Leu), is not present in control populations (gnomAD v4.1.0), having been previously reported in ClinVar 4 times (initially classified once as Variant of Uncertain Significance, and subsequently 3 times as Likely Pathogenic). [1] The variant is reported as Likely Pathogenic in LOVD (Leiden Open Variation Database) in a 66-year-old patient exhibiting limb-girdle muscular dystrophy with late onset (#0000172682). The suggested classification by several platforms (hg38 InterVar, GeneBe, Varsome, Franklin by Genoox) is Pathogenic/Likely Pathogenic. Moreover, *in silico* prediction tools, such as CADD, MPA, PolyPhen2, SIFT and MutationTaster, consistently predict that the described variant is likely to have a damaging or deleterious effect on protein integrity, further suggesting a pathogenic nature. Segregation studies were not possible as the patient’s parents are deceased. |
| Protein | The Pro334 residue is localized in the second hinge domain of the ACTA1 monomer, allowing intramolecular movements (Supplementary Figure 3A). [2] It is highly conserved in 11 species, being found in a highly conserved region of the protein (Supplementary Figure 3B).  Replacing proline with leucine, a more flexible and hydrophobic amino acid, could have significant implications, as proline and leucine differ in structure and properties, and the hinge region is crucial for actin's flexibility and function (Supplementary Figure 3C). Indeed, proline's rigid cyclic structure is essential for maintaining the structural constraints and flexibility of the hinge, probably facilitating actin's interactions with myosin and other regulatory proteins.  Although residue 334 does not seem directly involved in interactions between actin monomers during polymerisation (Supplementary Figure 4), it could play a role in interactions with other actin filament-associated proteins such as troponin, tropomyosin, or nebulin, which are essential for the regulation and stabilisation of filament structure and function. |
| cardiac alpha-actin expression | Immunostaining using anti-ACTC1 antibody highlighted an overexpression of cardiac muscle alpha actin in the patient’s biopsy compared to controls. This observation was confirmed by western blot analysis. We could also observe a significant fibre hypertrophy in the patient’s muscle biopsy compared to the control. |

1. National Center for Biotechnology Information. ClinVar; [VCV000532770.28], <https://www.ncbi.nlm.nih.gov/clinvar/variation/VCV000532770.28> (accessed Jan. 27, 2025).

2. Sparrow, J.C., et al., *Muscle disease caused by mutations in the skeletal muscle alpha-actin gene (ACTA1).* Neuromuscular Disorders, 2003. **13**(7): p. 519-531.
